# Efficacy of anti-calcitonin gene-related peptide monoclonal antibodies in hemiplegic migraine: a case report and review of literature

**DOI:** 10.3389/fneur.2025.1579203

**Published:** 2025-04-08

**Authors:** Máté Héja, László Oláh

**Affiliations:** Department of Neurology, Faculty of Medicine, University of Debrecen, Debrecen, Hungary

**Keywords:** migraine, hemiplegic migraine, CGRP, monoclonal antibody, fremanezumab

## Abstract

Hemiplegic migraine (HM) is a rare subtype of migraine with aura characterized by transient unilateral motor weakness during attacks. Although monoclonal antibodies (mABs) targeting the calcitonin gene-related peptide (CGRP) pathway have shown efficacy in migraine prevention, their role in HM remains largely unexplored, since these patients are generally excluded from randomized clinical trials aimed at developing migraine preventive drugs. We present a case of a middle-aged woman with chronic migraine and recurrent hemiplegic episodes treated with fremanezumab. After 11 months of monthly 225 mg subcutaneous fremanezumab injections, the patient experienced a substantial reduction in monthly headache days, aura episodes, and symptom severity, without safety concerns. This case adds to the emerging evidence supporting CGRP mABs as a potential therapeutic option for HM. Further research is needed to elucidate their precise mechanism and determine their efficacy in broader HM populations.

## 1 Introduction

Migraine is one of the most prevalent and disabling neurological disorders characterized by recurrent attacks of moderate or severe throbbing headache, typically unilateral and frequently accompanied by nausea and sensitivity to light and sound. According to the Global Burden of Disease study in 2019, migraine is the second leading cause of disability, and first among women aged 15 to 49 (1). One-third of migraineurs experience aura symptoms, which are integral parts of the migraine episodes and consist of a group of transient focal neurological symptoms that last from 5 to 60 min and usually resolve prior to or in the early phase of a migraine headache. The current criteria of the International Classification of Headache Disorders (ICHD-3) distinguish between typical aura (visual, sensory, speech or language disturbances) and atypical aura with brainstem symptoms, in addition to hemiplegic and retinal forms (2). Hemiplegic migraine (HM) is a rare subtype of migraine with aura including motor weakness. To diagnose HM, the following criteria must be met: (1) at least two attacks fulfilling the criteria for migraine with aura; (2) fully reversible motor weakness; (3) fully reversible visual, sensory or speech/language symptoms (2). The presence of both headaches and aura symptoms in HM significantly increases the burden on patients, and the management of HM can be challenging. Furthermore, patients with HM represent a significant portion of stroke mimics and may lead unnecessary and potential harmful treatments (e.g., thrombolysis) (3). Monoclonal antibodies (mABs) targeting the calcitonin gene-related peptide (CGRP) pathway represent a novel treatment approach in migraine prevention. Currently, there are four mABs approved as migraine preventive treatment: galcanezumab, eptinezumab and fremanezumab are antibodies that target CGRP ligand, whereas erenumab targets CGRP receptor. They all have demonstrated efficacy, safety and tolerability in multiple randomized clinical trials in adults with both episodic and chronic migraine (4) and are thus recommended for migraine prevention in the latest guidelines (5); however, the literature regarding their effectiveness in HM is scarce. Here, we present a case where fremanezumab was successfully used to treat a patient with HM in our tertiary headache center, and review the available literature on the topic.

## 2 Case description

A 53-year-old smoker female patient with well-treated hypertension, bronchial asthma, mixed anxiety-depressive disorder and goiter was admitted to our Neurological Intensive Care Unit in December 2021 as a stroke-alert with sudden onset of left sided weakness and facial droop. The patient reported that at the onset of the symptoms, she experienced numbness in the left-sided limbs, followed by difficulties with speech. Approximately 20 min later, an intense, throbbing headache similar to previous episodes started on the right side, accompanied by weakness in the extremities on the left. On admission, her neurological examination revealed moderately severe left sided hemiparesis and central facial palsy (NIHSS: 6 points) accompanied by mild occipital headache, paresthesia in the left upper and lower limb, nausea and dizziness of varying intensity. Non-contrast cerebral computed tomography (NCCT) showed no signs of bleeding or acute ischemic lesion, CT-angiography did not reveal occlusion or significant stenosis on the major cervical and intracranial arteries. Based on the clinical symptoms, right hemispheric ischemic stroke was suspected and intravenous thrombolysis was started.

The patient’s neurological status did not change during thrombolysis however, by the next day, her neurological symptoms and accompanying complaints had resolved. CT scan performed 24-h after the onset of symptoms was negative. Routine cerebrovascular check-up (blood profile, carotid ultrasound, transthoracic echocardiography) did not reveal any abnormality. Detailed medical history was taken, during which it was revealed that about a year before, the previously infrequent headaches became much more frequent. According to the patient (prior to this event, she did not keep a headache diary), the headache was unilateral, throbbing type, moderate or severe intensity, had temporoparietal localization, and accompanied by nausea, vomiting and light sensitivity 3–5 times per week. Nonsteroidal anti-inflammatory drugs (NSAIDs), such as ibuprofen and diclofenac failed to control these attacks. The number of monthly migraine days (MHDs) in the past 3 months had exceeded 20. Since the number of headache days exceeded 15 days per month for more than 3 consecutive months, which, on at least 8 days per month, had the features of migraine headache, the patient fulfilled the diagnostic criteria for chronic migraine (2). She used over-the-counter painkillers on a daily basis, therefore, medication overuse headache (MOH) also worsened the patient’s condition. Her family history was negative in terms of migraine, she had no siblings or children. Sodium valproate was initiated for prophylaxis, in addition to low-dose aspirin for secondary stroke prevention. We encouraged the patient to keep a headache diary.

Nine months later, the patient was admitted to our department again due to headache accompanied by left-sided weakness and facial asymmetry within the thrombolytic time window. According to the patient’s report, the sequence of symptom onset was identical as described above. Upon admission, physical examination revealed mild left-sided hemiparesis, left sided central facial palsy and varying degrees of motor aphasia (NIHSS: 5 points). NCCT and CT-angiography were negative. Since the patient had disabling symptoms, an ischemic stroke could not be ruled out, she was within the thrombolytic time window, and there were no contraindications to reperfusion therapy, systemic thrombolysis was performed. Despite the treatment the hemiparesis persisted the following day and resolved gradually the day after. Analgetic infusions were needed multiple times to decrease the intensity of headache. Follow-up CT scan did not show any ischemic lesion or bleeding. Given the patient’s young age and the recurrence of stroke events within a short period of time, a thorough cerebrovascular investigation was conducted. Transoesophageal echocardiography (TEE) did not reveal any source of cardiogenic emboli. 24-h electrocardiogram monitoring did not detect arrhythmias. Magnetic resonance imaging (MRI) of the brain showed two T2 and FLAIR hyperintense foci in the right frontal subcortical region without any clinical significance. Electroencephalography (EEG) showed generalized beta activity without any epileptiform discharges (the patient was taking benzodiazepines regularly due to psychiatric comorbidity). Thrombophilia screen test did not reveal any evidence of inherited deficiencies of naturally occurring anticoagulant factors, or antibodies associated with anti-phospholipid syndrome. Platelet aggregation test showed complete inhibition on low-dose aspirin treatment. Examination of the cerebrospinal fluid (CSF) showed normal cell count, mildly elevated protein concentration (60.5 mg/dL) and normal immunoglobulin G level with polyclonal pattern. Lactate stress test ruled out mitochondrial disease and family history was also negative. The patient stated that while taking the previously recommended sodium valproate, she experienced a skin rash and discontinued the medication. Based on the headache diary, she had 17 ± 1.15 (SD) MHDs on average, the average duration was 11.8 ± 7.8 h and average severity was 7.9 ± 1.7 from 10 according to the Visual Analog Scale (VAS) per occasion. Typical aura symptoms (mostly sensory) occurred once or twice per month during the attacks. Bothersome symptom occurred on average over 14 ± 1 days, and acute medication use occurred on average over 14.3 ± 1.2 days, therefore the criteria for MOH were met (2). She reached 29 points on Migraine Disability Assessment test (MIDAS). The recurring headache attacks with completely reversible motor symptoms, along with the migraine history, raised the possibility of hemiplegic migraine. Topiramate was started as a migraine preventive drug.

**Figure 1:**
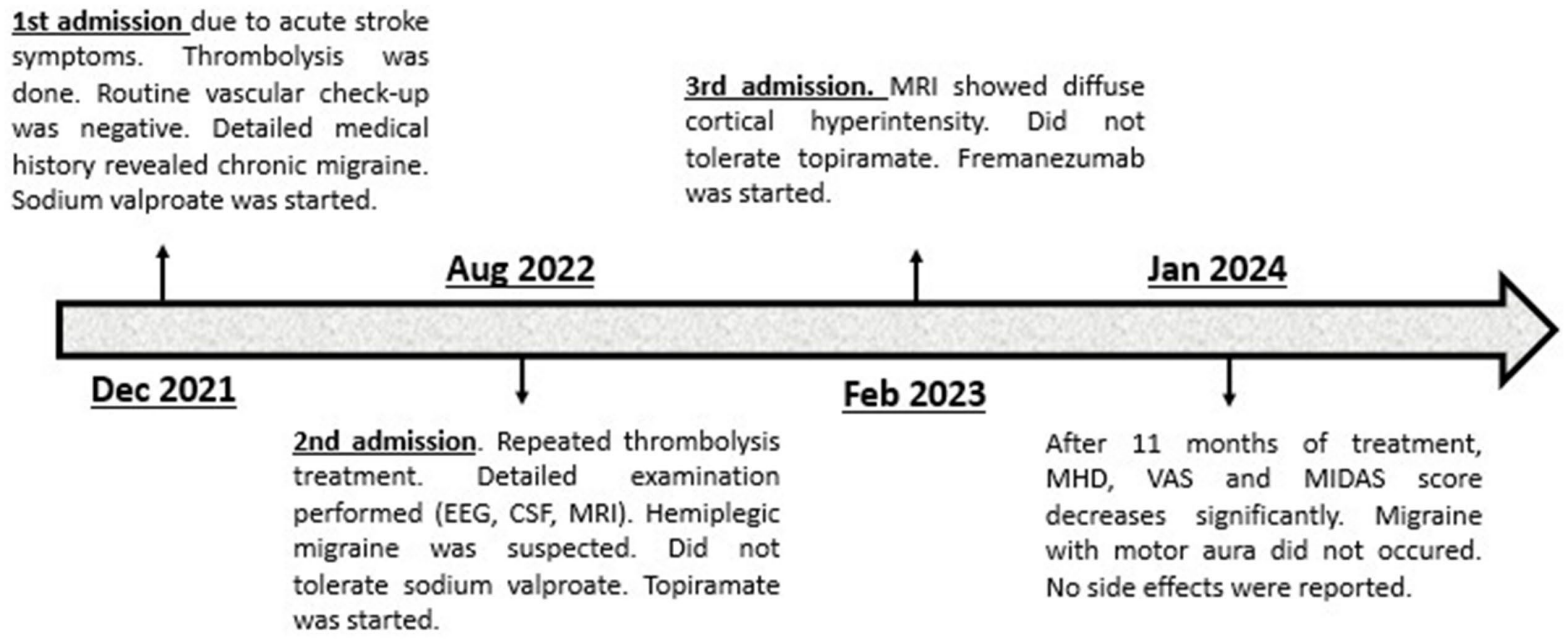
Timeline of patient’s case. EEG, Electroencephalography; CSF, Cerebrospinal fluid; MRI, Magnetic resonance imaging; MHD, Monthly headache days; VAS, Visual analog scale; MIDAS, Migraine disability assessment.

Another six months later, the patient was again transported to our department by paramedics due to symptoms practically identical to the previous ones. Upon admission, in her neurological status left sided central facial palsy, severe left upper extremity paresis, moderately severe left lower extremity paresis, left sided paresthesia and tactile hypesthesia and motor aphasia were seen. An urgent brain MRI was performed, which showed diffuse cortical hyperintensity on T2, FLAIR and DWI sequences without any signs of hyperacute stroke (Supplementary Figure S1). Therefore, thrombolysis was not performed. We administered 1,000 mg of paracetamol infusion accompanied by antiemetics to treat the patient’s migraine headache and as a result, her neurological symptoms and the headache improved; 150 however, mild hemiparesis was still seen on the following day. EEG and CSF examination were repeated with negative results. Magnetic evoked potential (MEP) was performed and it showed decreased cortical amplitudes bilaterally suggesting intracranial pyramidal tract lesion. The patient 153 stated that topiramate caused unbearable dizziness and daytime drowsiness, therefore, after a few 154 weeks, she stopped taking the medication. The headache parameters did not change over the past 155 period. Since the patient did not tolerate two preventive medications, a trial of fremanezumab with a 156 monthly 225 mg subcutaneous injection was proposed. After being informed of the potential risks and 157 current evidence, the patient provided consent. At 12 weeks of treatment, the number of MHDs 158 decreased to 3 or 4 with shorter duration, less severe intensity and without or less bothersome 159 accompanying symptoms. Follow-up MRI showed complete regression of cortical hyperintensity. 160 Currently, she is under treatment with fremanezumab for 11 months, and the efficacy particularly with 161 regards to frequency and duration of the headaches has been improving over time. According to the headache diary from her last visit to the clinic, MHDs, average duration, and average severity on VAS 163 were 3.6 ± 0.6 days, 2.2 ± 1.1 h, and 5.9 ± 1.2, respectively. Bothersome symptom occurred on average over 1.3 ± 0.6 days, and acute medication use occurred on average over 2 ± 1 days. Her MIDAS score was 5. Across the treatment period, no migraines with motor or other aura symptoms occurred. No side 166 effects were reported. Figure 1 presents the patient's brief history in the form of a timeline.

## 3 Discussion

HM is an uncommon subtype of migraine with aura characterized by the temporary weakness on one side of the body as aura manifestation in at least a few of the attacks. Although “plegia” means paralysis, in most cases, the attacks are characterized by weakness rather than complete paralysis; however, weakness can rarely be bilateral and sometimes it may switch/change side during the attacks (2). A key characteristic of HM is that the symptoms resolve completely; however, the weakness may outlast the headache and rarely can last up to 4 weeks (6). Since, there are no pathognomonic clinical, laboratory or radiological findings in HM, the diagnosis is primarily based on detailed history and the exclusion of other possible causes.

Here, we presented a case of a middle-aged woman with a history of chronic migraine and severe headache attacks accompanied by aphasic, sensory and motor aura, highly suspected for HM; however, at first glance, she presented a real differential diagnostic challenge. The ictal onset of focal neurological deficits, in the presence of vascular risk factors (hypertension, smoking), raised the impression of an acute stroke; therefore, the patient’s care was conducted according to the stroke protocol in every occasion. If the history and physical examination suggest ischemic stroke, the initial imaging rules out intracranial bleeding and the patient is otherwise eligible for reperfusion treatment, the general rule is that the worst should be assumed, and thrombolysis should be performed. Numerous case studies have already demonstrated that HM can mimic a stroke when attacks have an abrupt onset and aura is prolonged (7, 8) and, as in our case, it may lead to unnecessary thrombolytic therapy. To avoid the misdiagnosis, it is important to describe the correct order and duration of the symptoms, to take a detailed family history and to perform an appropriate clinical examination. Although our patient’s family history was negative for migraine, we had a strong suspicion of hemiplegic migraine due to the following reasons: recurrent, stereotyped and transient episodes of sensory-motor deficits followed or accompanied by headache attack with features of migraine, history of chronic migraine, normal CT and MRI scan, and negative thorough cerebrovascular investigation.

The pathomechanism of HM is not fully understood and involves both genetic and neurovascular factors. Familial hemiplegic migraine (FHM), an autosomal dominant hereditary form of HM, is linked to mutations in specific genes (CACNA1A, ATP1A2, SCN1A) that encode ion channels and transporters critical for neuronal excitability and synaptic transmission. These mutations disrupt ionic homeostasis, increasing cortical excitability and susceptibility to cortical spreading depolarization (CSD) (9). CSD is considered the underlying mechanism for the aura phase of migraine, including the motor aura in HM (10). In FHM, the genetic mutations enhance the brain’s vulnerability to CSD, contributing to transient neurological deficits such as hemiparesis. Sporadic hemiplegic migraine (SHM) shares similar pathophysiological mechanisms with FHM but lacks a clear familial inheritance pattern. Environmental triggers and acquired alterations in neuronal excitability may play a more prominent role in SHM (11).

Over the past 30 years, both clinical and preclinical studies have proven that CGRP has an essential role in migraine pathophysiology (12). CSD can trigger the release of CGRP in a calcium-dependent manner (13), and blocking CGRP receptors has been shown to suppress CSD-induced arterial dilation and plasma protein extravasation, suggesting a role of CGRP in CSD-related vascular changes (14). Understanding the interplay between CGRP and CSD has led to the development of CGRP-targeted therapies. However, the role of CGRP in HM is not well-established and has been a subject of research. A study conducted by Hansen et al. on 9 genetically confirmed FMH patients showed that CGRP infusion did not induce aura symptoms in any of the participants and there were similar rates of reported migraine-like headaches compared to healthy control group (15). A similar Danish study on 11 FMH patients with no identified gene mutations found no difference in terms of reported migraine-like attacks and similar rates of post-infusion headache compared to healthy individuals (16). These findings suggested that the pathophysiologic pathways underlying headache and aura in FHM may be different from the common types of migraine and raised questions about the effectiveness of CGRP antagonists in treating FHM.

Since, there are no randomized controlled trials in patients with HM, the current therapeutic recommendations are based on case reports and small studies which have suggested that calcium channel blockers, such as verapamil and flunarizine, acetazolamide and certain antiepileptic drugs, such as lamotrigine, valproic acid and topiramate may be effective as a preventive medication (6). While early research questioned the role of CGRP in HM, more recent studies indicated that CGRP targeted therapies may offer benefits to some HM patients. Danno et al. reported a case series on six HM patients, four with SHM and two with FMH, treated with galcanezumab, a monoclonal antibody targeting the CGRP-pathway. After 3 months of treatment, three patients experienced a reduction in the number of monthly headache days of at least moderate severity, motor weakness decreased in four of the six patients and disappeared in two of these patients, and improvement in MIDAS score was observed in five of the six participants. None of the patients reported adverse effects (17). More recently, D’Apolito et al. reported a case of a woman with frequent migraine attacks, preceded by aphasic, sensory and motor aura, highly suspected for HM, with a novel missense mutation in the SCN1A gen. The attacks were unresponsive to NSAIDs and triptans, and preventive therapy with topiramate and amitriptyline was unsuccessful. After three doses of galcanezumab she experienced great improvement of headache frequency and intensity, which was persistent at one-year follow up, moreover, the patient did not report any hemiplegic aura episodes (18). In a very recent case study, Antenucci et al. reported a young male patient with SHM carrying a CACNA1A mutation with frequent headache attacks accompanied by visual, sensory and motor aura. The attacks showed minimal response to NSAIDs, with requiring an average intake of 19 tablets a month, while traditional preventive medications (e.g., topiramate, lamotrigine, amitriptyline) were either ineffective or poorly tolerated. After three doses of eptinezumab, the patient demonstrated significant improvement in headache and aura frequency with a reduction of NSAID use and MIDAS score. At the six-month follow-up, the improvement remained consistent with a 58% reduction in headache days per month and a 67% decrease in motor aura attacks per month. No adverse events were documented (19).

It is worth emphasizing that, there are similarities between our patient and the previously reported cases. Firstly, all of the patients experienced high-frequency, disabling migraine attacks with medication overuse, and they either failed at least two preventive medications or were intolerant to other therapies. The anti-CGRP treatment effectively improved all headache parameters, including frequency, intensity, NSAID usage, associated symptoms and MIDAS score, resulting in a significant improvement in the patient’s quality of life. According to our patient, the improvement of quality of life was the greatest success of the treatment. This supports the fact that CGRP antagonists can be effective even in such difficult-to-treat cases, without significant safety concerns. Secondly, not only did the headache improve, but the patients demonstrated a beneficial effect on aura recurrence also, both typical and motor auras. To our knowledge, this is the first presented case of a positive response to fremanezumab in a patient with SHM. This observation provides additional evidence supporting the role of CGRP in the pathogenesis of aura in HM patients, however the exact mechanism is still unknown. CGRP mABs are large molecules that do not readily cross the blood–brain barrier (BBB) under normal conditions, and their primary mechanism of action is believed to be peripheral, targeting the trigeminovascular system (20). However, recent studies suggest that these antibodies may have indirect effects on central nervous system activity. For instance, research has shown that treatment with erenumab and galcanezumab can alter brain activity in areas such as the hypothalamus, the periaqueductal gray matter and secondary somatosensory cortex, which are involved in migraine pathophysiology (21, 22). In a recent cohort study conducted by Braca et al. the anti-CGRP mABs, including fremanezumab, were proven highly effective in migraine with aura, both in reducing mean monthly aura days and mean monthly days with headache. They hypothesized that by inhibiting the action of CGRP, anti-CGRP mABs can reduce neuronal excitability, suppress the release of other neurotransmitters associated with CSD, and decrease the brain tissue’s vulnerability to CSD (23). However, a recent study using animal models of migraine aura found that fremanezumab was able to slow the propagation of CSD but did not prevent its initiation, suggesting that other mechanisms are also involved in CSD initiation (24). Further studies are needed to provide a more comprehensive understanding of impact of anti-CGRP mABs on the pathophysiology of migraine with aura.

Since this is a case report, it is difficult to generalize our findings, which represents the main limitation of our work. Furthermore, no genetic testing was performed in our patient, primarily due to financial constraints, which limits the comparison of this case with previously reported FMH patients and sporadic cases with novel mutations. Another limitation, is that the follow-up period was only 11 months, which is comparable to the average attack frequency observed in patients with HM (1–3 attacks per year). Therefore, in order to clearly conclude the effectiveness of fremanezumab in HM, a longer follow-up is necessary. Nevertheless, this case report still provides additional evidence regarding the potential use of anti-CGRP mABs in the treatment of this rare migraine variant.

## 4 Conclusion

The diagnosis of HM is difficult due to its rarity and distinctive clinical presentation, which can mimic other, more common neurological conditions, such as acute stroke. Due to the limited evidence, there are no clear recommendations regarding the use of anti-CGRP mABs in HM. Our findings suggest that fremanezumab provides an effective and safe alternative therapeutic option in patients with high degree of disability and no response to conventional preventive treatments. Future studies are needed to determine whether anti-CGRP antibodies truly offer a valid option in HM, and to identify which patients are most likely to benefit from this treatment.

## Data Availability

The original contributions presented in the study are included in the article/Supplementary material, further inquiries can be directed to the corresponding author.

## Glossary

BBB: Blood–brain barrier
CGRP: Calcitonin-gene related peptide
CM: Chronic migraine
CSD: Cortical spreading depolarization
CSF: Cerebrospinal fluid
DWI: Diffusion-weighted imaging
FHM: Familial hemiplegic migraine
EEG: Electroencephalography
HM: Hemiplegic migraine
ICHD-3: International Classification of Headache Disorders-3
mAB: Monoclonal antibody
MIDAS: Migraine disability assessment
MEP: Magnetic evoked potential
MHD: Monthly headache days
MOH: Medication overuse headache
MRI: Magnetic resonance imaging
NCCT: Non-contrast cerebral computed tomography
NIHSS: National Institute of Health Stroke Scale
NSAID: Nonsteroidal anti-inflammatory drug
VAS: Visual analog scale

## References

[1] Steiner TJ Stovner LJ Jensen R Uluduz D Katsarava Z. Lifting the burden: the global campaign against headache. Migraine remains second among the world's causes of disability, and first among young women: findings from GBD2019. J Headache Pain. (2020) 21:137. doi: 10.1186/s10194-020-01208-0 33267788 PMC7708887

[2] IHS. Headache Classification Committee of the International Headache Society (IHS) The international classification of headache disorders, 3rd edition. Cephalalgia. (2018) 38:1–211. doi: 10.1177/0333102417738202 29368949

[3] Pohl M Hesszenberger D Kapus K Meszaros J Feher A Varadi I. Ischemic stroke mimics: A comprehensive review. J Clin Neurosci. (2021) 93:174–82. doi: 10.1016/j.jocn.2021.09.025, PMID: 34656244

[4] Haghdoost F Puledda F Garcia-Azorin D Huessler E-M Messina R Pozo-Rosich P. Evaluating the efficacy of CGRP mAbs and gepants for the preventive treatment of migraine: A systematic review and network meta-analysis of phase 3 randomised controlled trials. Cephalalgia. (2023) 43:366. doi: 10.1177/03331024231159366, PMID: 36855951

[5] Puledda F Sacco S Diener H-C Ashina M al-Khazali HM Ashina S. International headache society global practice recommendations for preventive pharmacological treatment of migraine. Cephalalgia. (2024) 44:735. doi: 10.1177/03331024241269735, PMID: 39262214

[6] Kumar A Samanta D Emmady PD Arora R. Hemiplegic migraine In: StatPearls. Treasure Island, FL Arora R (Ed.). Forest Hills, NY: StatPearls Publishing (2024) 30020674

[7] Kaushal R Kashyap A Yogesh S Agarwal M Banerjee I. A rare case of sporadic hemiplegic migraine mimicking stroke: A diagnostic challenge solved by comprehensive history taking. Cureus. (2024) 16:e57790. doi: 10.7759/cureus.57790, PMID: 38721208 PMC11076926

[8] Ryan DJ Coughlan S McSweeney N Dineen J Fanning N. Hemiplegic migraine as a stroke mimic: imaging and electroencephalography findings. Stroke. (2023) 54:e306–9. doi: 10.1161/STROKEAHA.122.041369, PMID: 37194626

[9] Russell MB Ducros A. Sporadic and familial hemiplegic migraine: pathophysiological mechanisms, clinical characteristics, diagnosis, and management. Lancet Neurol. (2011) 10:457–70. doi: 10.1016/S1474-4422(11)70048-5, PMID: 21458376

[10] Goadsby PJ. Pathophysiology of migraine. Ann Indian Acad Neurol. (2012) 15:15–22. doi: 10.4103/0972-2327.99993, PMID: 23024559 PMC3444225

[11] Di Stefano V Rispoli MG Pellegrino N Graziosi A Rotondo E Napoli C. Diagnostic and therapeutic aspects of hemiplegic migraine. J Neurol Neurosurg Psychiatry. (2020) 91:764–71. doi: 10.1136/jnnp-2020-322850, PMID: 32430436 PMC7361005

[12] Edvinsson L Goadsby PJ. Discovery of CGRP in relation to migraine. Cephalalgia. (2019) 39:331–2. doi: 10.1177/0333102418779544, PMID: 30827155

[13] Tozzi A de Iure A Di Filippo M Costa C Caproni S Pisani A. Critical role of calcitonin gene-related peptide receptors in cortical spreading depression. Proc Natl Acad Sci. (2012) 109:18985–90. doi: 10.1073/pnas.1215435109, PMID: 23112192 PMC3503217

[14] Schain AJ Melo-Carrillo A Stratton J Strassman AM Burstein R. CSD-induced arterial dilatation and plasma protein extravasation are unaffected by Fremanezumab: implications for CGRP's role in migraine with Aura. J Neurosci. (2019) 39:6001–11. doi: 10.1523/JNEUROSCI.0232-19.2019, PMID: 31127003 PMC6650995

[15] Hansen JM Thomsen LL Olesen J Ashina M. Calcitonin gene-related peptide does not cause the familial hemiplegic migraine phenotype. Neurology. (2008) 71:841–7. doi: 10.1212/01.wnl.0000325482 18779512

[16] Hansen JM Thomsen LL Olesen J Ashina M. Calcitonin gene-related peptide does not cause migraine attacks in patients with familial hemiplegic migraine. Headache. (2011) 51:54453. doi: 10.1111/j.1526-4610.2011.01861.x 21457239

[17] Danno D Ishizaki K Kikui S Takeshima T. Treatment of hemiplegic migraine with anticalcitonin gene-related peptide monoclonal antibodies: A case series in a tertiary-care headache center. Headache. (2023) 63:984–9. doi: 10.1111/head.14591 37366160

[18] D'Apolito M Rispoli MG Ajdinaj P Travaglini D Bonanni L. Sporadic hemiplegic migraine with novel missense mutation in the SCN1A gene and positive response to anti-CGRP antibody: a case report. Neurol Sci. (2024) 45:5535–7. doi: 10.1007/s10072-024-07665-8, PMID: 38940877

[19] Antenucci P Pes F Cesnik E Capone JG Padroni M. A case of sporadic hemiplegic migraine treated with eptinezumab: should we consider anti-CGRP antibodies for selected patients? Neurol Sci. (2025) 45:5535–5537. doi: 10.1007/s10072-024-07980-0, PMID: 39745588

[20] Edvinsson L. CGRP receptor antagonists and antibodies against CGRP and its receptor in migraine treatment. Br J Clin Pharmacol. (2015) 80:193–9. doi: 10.1111/bcp.12618 25731075 PMC4541967

[21] Basedau H Sturm LM Mehnert J Peng KP Schellong M May A. Migraine monoclonal antibodies against CGRP change brain activity depending on ligand or receptor target—an fMRI study. eLife. (2022) 11:e77146. doi: 10.7554/eLife.77146, PMID: 35604755 PMC9126581

[22] Ziegeler C Mehnert J Asmussen K May A. Central effects of erenumab in migraine patients: an event-related functional imaging study. Neurology. (2020) 95:e2794–802. doi: 10.1212/WNL.0000000000010740, PMID: 32917805

[23] Braca S Miele A Stornaiuolo A Cretella G de Simone R Russo CV. Are anti-calcitonin gene-related peptide monoclonal antibodies effective in treating migraine aura? A pilot prospective observational cohort study. Neurol Sci. (2024) 45:1655–60. doi: 10.1007/s10072-023-07241-6, PMID: 38091211

[24] Melo-Carrillo A Schain AJ Stratton J Strassman AM Burstein R. Fremanezumab and its isotype slow propagation rate and shorten cortical recovery period but do not prevent occurrence of cortical spreading depression in rats with compromised blood-brain barrier. Pain. (2020) 161:1037–43. doi: 10.1097/j.pain.0000000000001791, PMID: 31895266 PMC7166155

