# Supplementary figures and images for: Efficacy of anti-calcitonin gene-related peptide monoclonal antibodies in hemiplegic migraine: a case report and review of literature
Source: Front Neurol. 2025 Apr 8;16:1579203. doi: 10.3389/fneur.2025.1579203 (PMC12011815; doi:10.3389/fneur.2025.1579203)

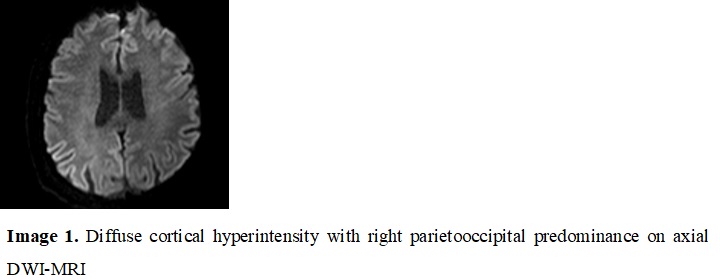

Supplement: Supplementary file 1 [file Image_1.JPEG]
